# External Cesium-137 doses to humans from soil influenced by the Fukushima and Chernobyl nuclear power plants accidents: a comparative study

**DOI:** 10.1038/s41598-020-64812-9

**Published:** 2020-05-13

**Authors:** Ka-Ming Wai, Dragana Krstic, Dragoslav Nikezic, Tang-Huang Lin, Peter K. N. Yu

**Affiliations:** 1 Department of Civil and Environmental Engineering, College of Engineering, Shantou University, Shantou, China; 2 Intelligent Manufacturing Key Laboratory of Ministry of Education, Shantou University, Shantou, China; 3 Faculty of Science, University of Kragujevac, R. Domanovica 12, Kragujevac, 34000 Serbia; 4 Center for Space and Remote Sensing Research, National Central University, Taoyuan City, Taiwan; 5 Department of Physics, City University of Hong Kong, Hong Kong SAR, China

**Keywords:** Biophysics, Cancer

## Abstract

External exposure to gamma-photon irradiation from soil contamination due to nuclear power plant (NPP) accidents has significant contribution to human radiation exposure in the proximity of the NPP. Detailed absorbed doses in human organs are rarely reported in the literature. We applied the Monte Carlo Neutron Particle (MCNP) transport code to calculate and compare the absorbed doses in different human organs. The absorbed doses by gamma-photon radiation were from cesium-137 (^137^Cs) in soil contaminated by the two major NPP accidents. More serious and wide-spread impacts of the Chernobyl NPP accident on soil contamination in Ukraine, Belarus, Russia and countries as far as Sweden and Greece were due to the inland location, radiative plume transport pathway and high ^137^Cs emission strength (9 times the Fukushima emission). Based on our MCNP calculations, the largest absorbed dose was found in skin. The maximum calculated external ^137^Cs annual effective dose received from the Chernobyl accident was 10 times higher relative to the Fukushima accident. Our calculated effective doses at various influenced areas were comparable to those available in the literature. The calculated annual effective doses at areas near the Fukushima and Chernobyl NPPs exceeded the ICRP recommendation of 1 mSv yr^−1^.

## Introduction

The Fukushima and Chernobyl nuclear power plant (FNPP and CNPP) accidents are the two largest sources of anthropogenic radionuclides released into the environment in recent years. The former was triggered by the Tohoku-earthquake induced tsunami on 11 March 2011, which caused damage of the main cooling systems and left the reactors overheated and led to hydrogen gas explosions. The explosions resulted in damaging of the FNPP buildings. The latter accident was initiated by an operating error on 26 April 1986 which caused xenon poisoning (and its reaction) of the Unit 4 reactor of the CNPP. It led to thermal destruction of the reactor and caused ignition of the graphite moderators. Following about one-quarter of the total radioactive materials released during the early stages of the accident, a second stage of the continuous release was due to the graphite fire^1^. The major difference between the two accidents is that the release of the FNPP is mainly chemical in nature (gas-phase only) but the CNPP released part of the core reactor inventory. Due to the adverse impacts of the CNPP accident to humans, a large-scale human evacuation from the CNPP was required (https://www-pub.iaea.org/MTCD/Publications/PDF/te_1240_prn.pdf) and resulted in a land use change. The land use change before and after the CNPP accident was detected through satellite observations (Supplementary Materials S1).

Specifically, cesium−137 (^137^Cs) with a half-life of 30.1 years derived from the NPP accidents has been intensively studied due to its significant adverse impacts on the environment through atmospheric deposition. The ^137^Cs deposition is depended on various factors such as strength/mode of accidental release, atmospheric transport pathways and plume inception in areas with local precipitation. Previous relevant studies have described the long-range transport of ^137^Cs from the CNPP in Ukraine affected European countries more than 1000 km away^1–4^. Similarly, local/regional and cross-Pacific transport of ^137^Cs and its downwind impacts from the FNPP accident has also been studied^5–10^. Other studies reported the measurements of ^137^Cs deposition after the atmospheric transport^11–24^.

The external radiation doses in the human body irradiated by gamma photons from ^137^Cs deposited in soil are of particular concern. Recent studies related to the FNPP accident for areas within the Fukushima prefecture, Japan have been undertaken^25,26^. Similar investigations at Bryansk, the most impacted areas by the CNPP accident in Russia, have been reported^27–29^. A study was carried out which focused on recent external doses in Minsk and Gomel, Belarus and Chernobyl, Ukraine in 2012^30^. It reported the estimated effective doses around CNPP to be still over the public dose limit of 1 mSv yr^−1^ recommended by the ICRP^31^ 26 years after the accident. The above studies were mainly undertaken at different local/regional-scale areas. A large-scale or country-scale comparative study on the soil distribution of ^137^Cs and associated absorbed doses in the human organs between different impacted areas are rarely available in the literature, despite the fact that the determination of organ doses is important for cancer risk assessment^32,33^. The organ doses estimation has been conducted for populations near nuclear facilities^34^.

In the current study, we first summarized, compared and explained the literature values of the ^137^Cs activity concentrations in soil measured in various areas influenced by the NPP fallouts. The external gamma-photon absorbed doses in different human organs due to derived ^137^Cs concentrations in contaminated soil in these areas were then calculated by the MCNP code. The calculated human organ doses (in more than ten organs) around the NPPs were then compared, which is the novelty of our study. Finally, we evaluated our calculated annual effective doses with the reported values available in the literature for the FNPP and CNPP accidents. Given the available soil concentrations of ^137^Cs, our study provided a method to calculate the annual effective doses at various areas where there were no estimates on effective doses available. The CNPP accident results were considered for the time immediately after the accident in order to have a meaningful comparison.

## Results and discussion

### Comparison of ^137^Cs soil concentrations due to the NPP accidents

Table 1 summarizes the literature values of ^137^Cs concentrations (kBq kg^−1^) in soil at various locations influenced by the FNPP and CNPP accidents. All the data reported here for CNPP accident are the values as in 1986. Not only the source-receptor distance, but also the meteorological setting for the radiative plume transport and soil property could affect the soil concentrations at a specific location. For the Fukushima event, the maximum soil concentration (66.7 kBq kg^−1^) was found in Minamisohma city 15 km from the NPP. Cities locating relatively far away from the NPP (>50 km) had smaller contamination of order of 10 kBq kg^−1^ or less. The data of ^137^Cs soil concentrations influenced by the CNPP accident were difficult to obtain since the available data in the literature were reported in kBq m^−2^. Extremely high soil concentration (>250 kBq kg^−1^) was found less than 30 km from the CNPP^14,18^. Within 100 km from the CNPP, the concentration was more than 20 kBq kg^−1^ but the contamination pattern was highly irregular and anisotropic. Total areas of 7200 and 116000 km^2^ with maximum concentrations of 23 and 2.8 kBq kg^−1^ were located in Belarus, Russia and Ukraine^16^. For the far field, Sweden, Austria and Greece showed abnormally high soil contamination^14^, attributed to the long-range transport of radiative plume. It was noted that the ^137^Cs deposition on Sweden was highly uneven^11^ and reference therein, and therefore the concentrations at both lower and higher sides were presented in Table 1. The uneven deposition was a result of changing meteorological conditions during the dispersion of released materials^14^.

**Table 1:** Summary of soil ^137^Cs concentrations contaminated by the FNPP and CNPP accidents.

Location	Distance from NPP (km)	Concentration (kBq kg^−1^)^*^	References
**FNPP** accident			
Namie town	8	33.1	^23^ (Table 1), ^24^ (Table S1)
Minamisohma city	15	66.7	^23^ (Table 1), ^24^ (Table S1)
J Village	20	9.4	^20^ (Table 1)
Motomiya city	50	8.4	^23^ (Table 1), ^24^ (Table S1)
Fukushima city	60	15.8	^21^ (Fig. 4), ^22^ (Fig. 1), ^23^ (Table 1), ^24^ (Table S1)
Koriyama city	60	11.4	^22^ (Fig. 1), ^23^ (Table 2), ^24^ (Table S1)
Nishi-Shirakawa county	85	1.6	^22^ (Fig. 1), ^24^ (Table S1)
**CNPP** accident			
Chistogalovka, Ukraine	3	87.1^****^	^17^ (Table 1)
Chernobyl	8	284.6^**^	^14^ (Plate 60)
Gomel Region, Belarus	30	480	^18^ (Fig. 2)
Outer area of Chernobyl and Gomel Region, Belarus	100	22.8^**^	^14^ (Plate 60)
Belarus, Ukraine, Russia (low concentration areas)	-^*****^	3.4^**, ****^	^16^ (Figs. 3–5)
Austria	1100	0.3^**^	^15^
Sweden (low concentration areas)	1250	0.3^**, ****^	^11^ (Fig. 1)
Sweden (high concentration areas)	1250	1.0^**, ****^	^11^ (Fig. 1)
Greece	1400	0.8^***^	^12^

*For the FNPP accident, arithmetic mean was reported when more than one reference reported the soil concentration for the same location. Concentrations at top soil (0–10 cm) were presented.

^**^A typical soil density of 1300 kg m^−3 (21)^ and top 5 cm sampled surface soil^11,17,30^ were assumed.

^***^A typical soil density of 1300 kg m^−3 (21)^ was assumed.

^****^Value corrected to 1 May 1986.

^*****^See text.

While some FNPP fallout occurred on the Japan landmass, the majority (80%) was on the northern Pacific Ocean along with the prevailing westerly^8,35,36^ since the FNPP is located at the coastline. Therefore the influences of the radiative plumes to other countries such as those in Northern America and Europe were small^7–10^. On the contrary, the CNPP was located well within the European continent. The large initial release height (>1 km above ground) of the radiative plumes due to explosions at the CNPP and convective updraft of the plumes during long-range transport were the major reasons of the high ^137^Cs deposition to areas in northern and southern European countries located >1000 km from the CNPP^1–3,37,38^. The large differences in soil contamination for the two accidents were also due to the large differences in atmospheric release of radionuclides ^137^Cs, which were in the range of 74–98 PBq for the CNPP^39–41^ and 12–17 PBq for the FNPP^7,42–44^.

### Calculated equivalent dose in organs and effective dose in areas influenced by the NPP accidents

**Figure 1:**
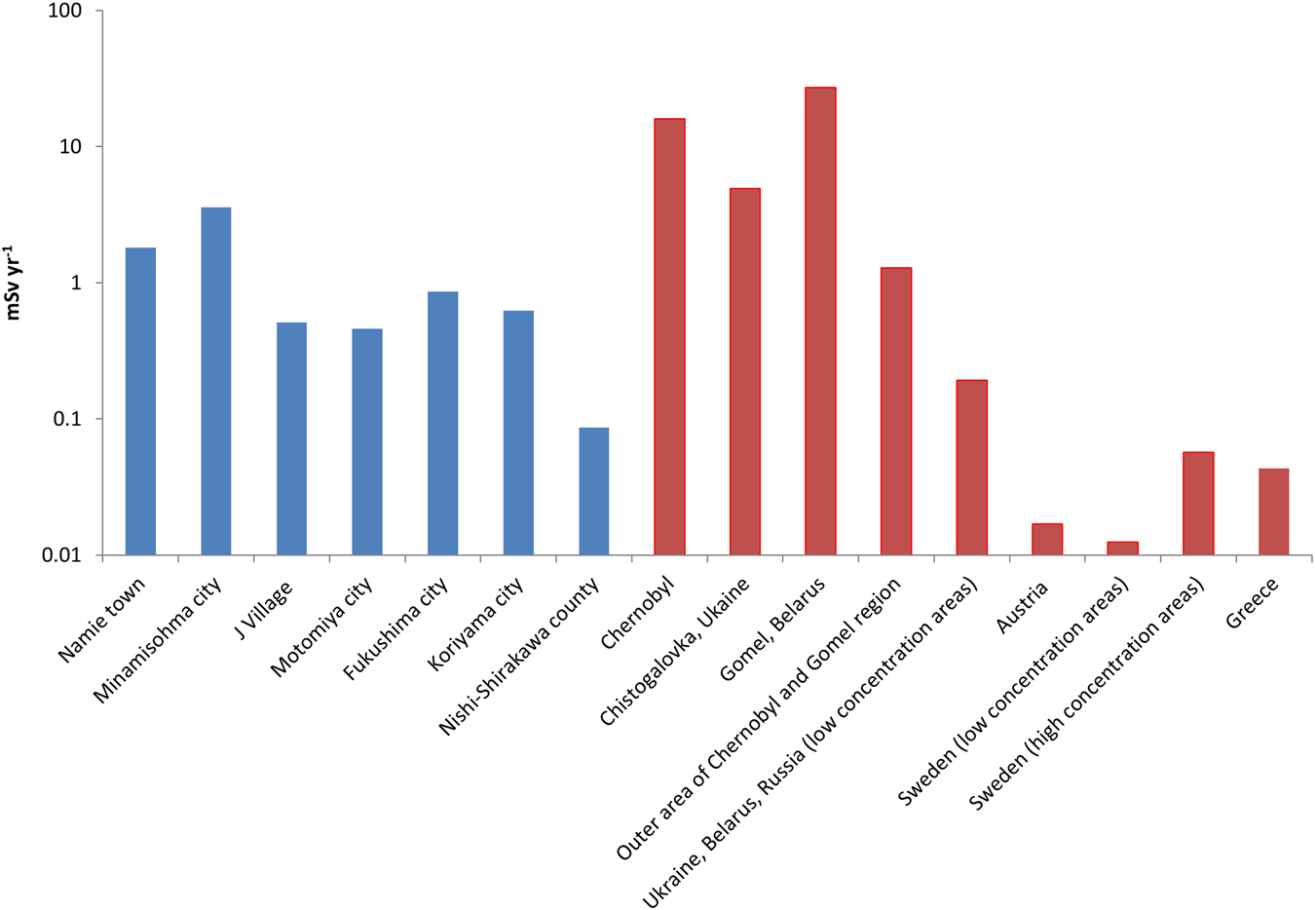
Comparison of external radiation doses from soil ^137^Cs. Annual external effective dose (mSv yr^−1^) calculated for various areas contaminated by the FNPP (blue) and CNPP (brown) accidents.

The MCNP modeling results calculated for equivalent doses in various organs of the mathematical phantom are given in Table 2. Skin and bone surface, and thyroid received the largest and smallest equivalent doses, respectively. Figure 1 shows the calculated annual external effective doses due to the ^137^Cs derived gamma photons irradiation calculated for various areas with contaminated soil influenced by the FNPP and CNPP accidents. The calculated annual effective doses for the CNPP accident were evaluated immediately after the accident as described above to have a meaningful comparison with the FNPP accident. The calculated maximum annual effective dose in areas affected by the CNPP accident was about 10 times higher compared with that in areas affected by the FNPP accident. Similarly, the near-field areas (in Ukraine, Belarus and Russia) received higher effective doses from the CNPP accident than those from the FNPP. Interestingly, the people habitat in Greece (~1400 km from the CNPP) received comparable effective dose as people in the Nishi-Shirakawa County, Japan located 85 km away from the FNPP. Even the lowest effective doses (0.01–0.02 mSv yr^−1^) calculated for Austria and Sweden for the current study, which were affected by the CNPP accident, were still several times higher than the background effective doses received in South Asian countries^45,46^.

**Table 2:** Calculated equivalent doses (µSv yr^−1^) in organs due to external dose from soil in various areas contaminated by the NPP accidents.

Location	Bone surface	Bone Marrow	Skin	Ovaries	Testes	Gonads	Breast	Lung	Thyroid	Liver	Bladder	Colon	Stomach	Esophagus	Remainder
**FNPP** accident															
Namie town	23000	6400	49000	1000	10000	5700	3200	2400	790	4300	6100	4400	3500	1700	8100
Minamisohma city	46000	13000	99000	2000	20000	11000	6400	4800	1600	8500	12000	8700	6900	3400	16000
J Village	6600	1800	14000	290	2900	1600	920	690	220	1200	1700	1200	1000	490	2300
Motomiya city	5900	1600	13000	260	2600	1400	820	620	200	1100	1500	1100	890	440	2100
Fukushima city	11000	3000	24000	480	4900	2700	1500	1200	380	2000	2900	2100	1700	830	3900
Koriyama city	8000	2200	17000	350	3600	2000	1100	840	270	1500	2100	1500	1200	600	2800
Nishi-Shirakawa county	1100	300	2400	48	500	270	160	120	38	210	290	210	170	83	390
**CNPP** accident															
Chernobyl	210000	57000	430000	8900	92000	51000	29000	22000	7000	38000	54000	39000	31000	15000	72000
Chistogalovka, Ukaine	63000	17000	130000	2700	28000	15000	8900	6700	2100	12000	17000	12000	9600	4700	22000
Gomel, Belarus	350000	96000	730000	15000	160000	85000	49000	37000	12000	64000	91000	66000	53000	26000	120000
Outer areas of Chernobyl and Gomel Region, Belarus	17000	4600	35000	720	7400	4100	2300	1700	560	3100	4300	3100	2500	1200	5700
Ukraine, Belarus, Russia with relatively low ^137^Cs concentration (2.8 kBq kg^−1^)	2500	680	5200	110	1100	610	350	260	84	460	650	470	380	190	860
Austria	220	60	460	9.4	97	53	31	23	7.4	40	57	41	33	16	75
Sweden (low concentration areas)	160	40	330	6.9	72	39	22	17	5.5	30	42	30	24	12	55
Sweden (high concentration areas)	730	200	1500	32	330	180	100	77	25	140	190	140	110	55	250
Greece	560	150	1200	24	250	140	79	59	19	100	150	110	85	42	190

For the accident in Japan, our result of calculated annual effective dose of 0.9 mSv yr^−1^ obtained for Fukushima City (Fig. 1) was well comparable to the external ^137^Cs dose assessment for the same City (0.5 mSv yr^−1^) by Yoshida and Suzuki^25^. Taira *et al*.^26^ reported the total external effective doses at the same location, which ranged from 2.2 to 7.6 mSv yr^−1^ after 3 month of the FNPP accident. Comparing with our dose calculations, higher dose levels were expected for the estimations by Taira *et al*. since they included the contribution from ^134^Cs radionuclides, which was higher (for example, the dose contribution of ^134^Cs was 3 times higher than ^137^Cs in the early period after the accident^47^). For the same reason, our calculated dose of 3.6 mSv yr^−1^ for the Minamisohma City was also comparable with that of 4.6 mSv yr^−1^ at a nearby village − Iitate Village^26^, to which ^134^Cs and ^137^Cs were the contributors. It is noted that the differences in dose levels could also be due to the adoption of different calculation methods. For instance, several empirical coefficients, such as occupancy-shielding factor, were adopted for the dose calculations for the work of Taira *et al*.

Regarding the accident in Ukraine, a recent study reported the external effective dose of 22 mSv yr^−1^ in soil samples taken in CNPP (Manany)^30^. The effective dose was calculated based on multiple radionuclides with predominated activity contribution from ^137^Cs. Our ^137^Cs dose calculation at the CNPP of 16 mSv yr^−1^ (Fig. 1) was thus reasonably compared with the reported value. There were large variations in the estimated external effective doses reported in the literature in western Bryansk, Russia - an area with the most significant soil contamination in Russia. For instance, Ramzaev *et al*.^28^ reported effective doses from 0.6 to 1.9 mSv yr^−1^ but Thornberg *et al*.^27^ reported values up to 2.8 mSv yr^−1^. The large variations in the effective doses were reflected by the corresponding heterogeneity in soil activity concentrations from <45 kBq m^−2^ to >1806 kBq m^−2^ ^16^. Nevertheless, our calculated effective dose for Russia in areas with low soil concentration (0.19 mSv yr^−1^ in Fig. 1) was comparable to the minimum dose (0.6 mSv yr^−1^) evaluated in these studies. Our result calculated for Gomel, Belarus (1.3 mSv yr^−1^, Fig. 1) was comparable to the estimation by Thornberg *et al*. (2.8 mSv yr^−1^)^27^, with similar soil contamination levels (≤1806 kBq m^−2^) in both Gomel and Bryansk^16^. All values presented here are corrected to values as in 1986.

## Methods

### Calculation of the conversion coefficients

**Figure 2:**
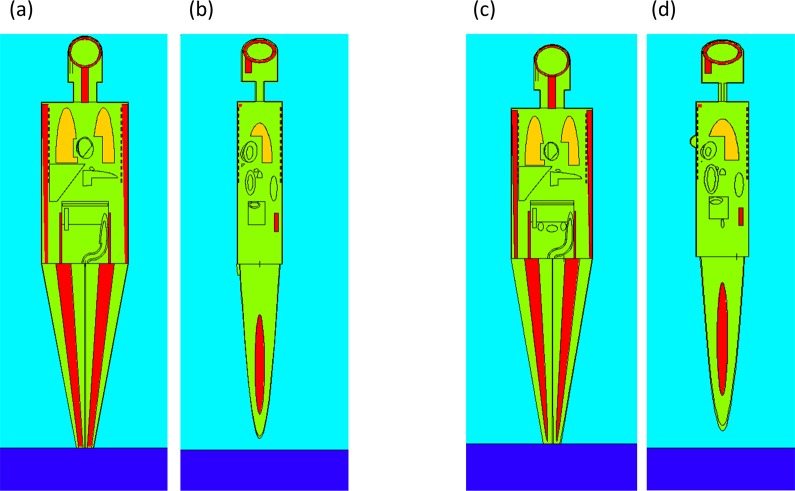
Mathematical phantoms output from the MCNPX code. (**a**) male (front-view); (**b**) male (side-view); (**c**) female (front-view); and (**d**) female (side-view).

The conversion coefficients for different human organs irradiated by gamma photons from ^137^Cs in soil were calculated by the MCNP5/X version 2.6.0 code^48^. Detailed descriptions of the coefficients for different organs are available elsewhere (e.g. Krstić and Nikezić^49^). Briefly, an Oak Ridge National Laboratory (ORNL) mathematical phantom described in ICRU Report 48^50^ and Eckerman *et al*.^51^ was adopted for the absorbed dose calculations^52^. Similar methods have been used for dose calculations in many studies^53–56^. The phantom consists of elliptical cylinders, truncated circular cones, circular cylinders, half ellipsoids, etc. to represent the trunk, arms, legs, feet, neck, head and female breasts. These “organs” were described by mathematical equations and inequalities^51^, which were then programmed in the input files for the MCNP code. Totally 66 (68) cells and 180 (188) surfaces were used for a male (female) phantom (Fig. 2). Transport of gamma photons with energy 662 keV from soil to target organs was simulated by the MCNP code. The soil source (pure SiO_2_) was assumed to be cylindrical shape with a radius of 3 m. Photons emitted farther than 3 m from the phantom have small probability to hit the target and were neglected here. The ^137^Cs vertical migration is known to be very slow and most of the accident-derived ^137^Cs was found within the top 15 cm of the soil^16^. The profiles reported in IAEA^16^ in Ukraine and Taira *et al*.^26^ in Iitate village, Japan were adopted respectively (Supplementary Materials S2) as the ^137^Cs distribution in soil cylinder impacted by the CNPP and FNPP accidents in the current study despite the information of site-specific ^137^Cs vertical profile in soil is not always available. The ^137^Cs concentration for the top 5 cm soil was about twice as that for the next 5 cm soil for both profiles. The soil profile adopted here is consistent with those reported elsewhere^57,58^. The cylinder representing the source was split into smaller cylinders 2 cm in height.

Homogenous distribution of the ^137^Cs radionuclides in smaller cylinders were assumed and uniform sampling of initial points in these small cylinders was applied. Totally, 10^8^ simulations were run for each source to ensure small calculation uncertainties (relative error is less than 10%). The MCNP energy deposition tally F6 was used for dose estimation. Conversion of units was applied to the MCNP results to obtain the conversion coefficient (in fGy per Bq s kg^−1^) for all major organs as a function of the source depth. The conversion coefficient is the absorbed dose rate in target organ per unit activity concentration in soil. The coefficient as a function of soil depth, which has been previously presented^49^, is given in Supplementary Materials S3.

### Calculation of the effective dose due to soil contamination from FNPP and CNPP accidents

The equivalent dose (H_t_) in a tissue or organ (t) was calculated through the sum:

1 $${H}_{t}=\sum _{r}{w}_{r}{D}_{t,r}$$

where *D*_*t,r*_ is the dose absorbed in that organ from ^137^Cs in soil for 1 year (in Gy y^−1^) from the radiation of type *r*; the radiation weighting factor is *w*_*r*_ = 1 for photons of all energies according to ICRP Publication 60^31^. The soil concentrations of ^137^Cs were obtained from Table 1 below. Contributions to the equivalent dose in organs from a soil layer were calculated by multiplying the above conversion coefficients with the number of disintegrations occurring during one year. The contributions from different layers were then summed up to obtain the equivalent dose in that organ.

The effective dose (E) for an individual standing on the ground was calculated according to ICRP Publication 116^59^:

2 $$E=\sum _{t}{w}_{t}({H}_{t,male}+{H}_{t,female})/2$$

where *H*_*t*,male_ and *H*_*t*,female_ are equivalent doses in male and female phantoms, respectively. The values of *w*_*t*_ are given in ICRP Publication 103^60^. An average outdoor occupancy factor of 0.3^61,62^ was included to the annual effective dose calculation. The factor used is comparable to the UNSCEAR’s value of 0.2, which is suggested to have differences around the world^63^. The differences are due to that, for instance, human is considered likely to spend more (less) time indoors for industrialized (agricultural) countries in temperate (warm) climates. More details of the occupancy factor could be referred to Hinrichsen *et al*.^64^ It is noted that the external dose is likely to be changed when the radionuclides migrate deeper into the soil^64^.

### Compilation of soil ^137^Cs concentration

The soil sample data were extracted from various references available in the literature as shown in Table 1. Concentrations in the top soil (0–10 cm) were presented. The mean concentrations were preferred. In case that the mean concentration was not available and there was a large variation of the concentration within a large area (e.g. Sweden), both lower- and higher-end concentrations were reported. For the data relevant to the FNPP accident, arithmetic means were reported when more than one reference reported the soil concentration for the same location. The data relevant to the CNPP accident were usually reported as kBq m^−2^. A typical soil density of 1300 kg m^−3 (21)^ and top 5 cm sampled surface soil^11,17,30^ were assumed if these information were not available.

## Concluding remarks

Our study summarized the literature values of ^137^Cs soil concentrations influenced by the CNPP and FNPP and rationalized their spatial distributions. The soil distributions were affected by the location of NPPs (either inland or coastal), initial release heights and emission strengths of the contaminants, as well as the atmospheric transport pathways. We then used these values and the conversion coefficients determined by the MCNP code to calculate the organs doses (µSv yr^−1^) due to ^137^Cs in soil in various areas contaminated by the NPP accidents, which are important for cancer risk assessment but were seldom reported. Finally, we discussed the similarity and differences of our calculated effective doses with values reported in the literature. Given the available soil concentrations of ^137^Cs, the annual effective doses at various locations could be calculated, where estimations of the effective doses at these locations were not available.

## Supplementary information

Supplementary Information 1.

Supplementary Information2.

Supplementary Information3.
