# Supplementary material for: External Cesium-137 doses to humans from soil influenced by the Fukushima and Chernobyl nuclear power plants accidents: a comparative study
Source: Sci Rep. 2020 May 13;10:7902. doi: 10.1038/s41598-020-64812-9 (PMC7220933; doi:10.1038/s41598-020-64812-9)
Supplement: Supplementary file 1 — Supplementary Information 1. [file 41598_2020_64812_MOESM1_ESM.pdf]

Supplementary Material S1

for

External Cesium-137 doses to humans from soil influenced by the Fukushima and Chernobyl nuclear power plants accidents: a comparative study

Ka-Ming Wai[1,2,*], Dragana Krstic[3], Dragoslav Nikezic[3], Tang-Huang Lin[4], Peter K.N. Yu[5,*]

[1]Department of Civil and Environmental Engineering, College of Engineering, Shantou University, Shantou, China

[2]Intelligent Manufacturing Key Laboratory of Ministry of Education, Shantou University, Shantou, China

[3]Faculty of Science, University of Kragujevac, R. Domanovica 12, Kragujevac 34000, Serbia

[4]Center for Space and Remote Sensing Research, National Central University, Taiwan

[5]Department of Physics, City University of Hong Kong, Hong Kong SAR, China

* Corresponding Authors

E-mail: jmwei@stu.edu.cn (Ka-Ming Wai)

E-mail: peter.yu@cityu.edu.hk (Peter K.N. Yu)

Supplementary Materials S1 Land-use change near the NPP observed by satellite

Figure S1 shows the land use change based on Normalized Difference Vegetation Index calculation[1] near the CNPP before and after the accident. Farmlands (i.e., yellow/light-green tiny areas with well-defined boundaries) near the CNPP before the accident were replaced by natural vegetation (i.e. same areas without well-defined boundaries) which was taken over the abandoned fields after the accident. The phenomenon happened in the areas within the "Chernobyl Exclusion Zone", 30 km from the CNPP, and even further. However, there was no affirmative land use change near the FNPP which could be attributed to the FNPP accident (figure not shown). It was partially due to the inability to resolve the small-scale farmlands by current image resolution.

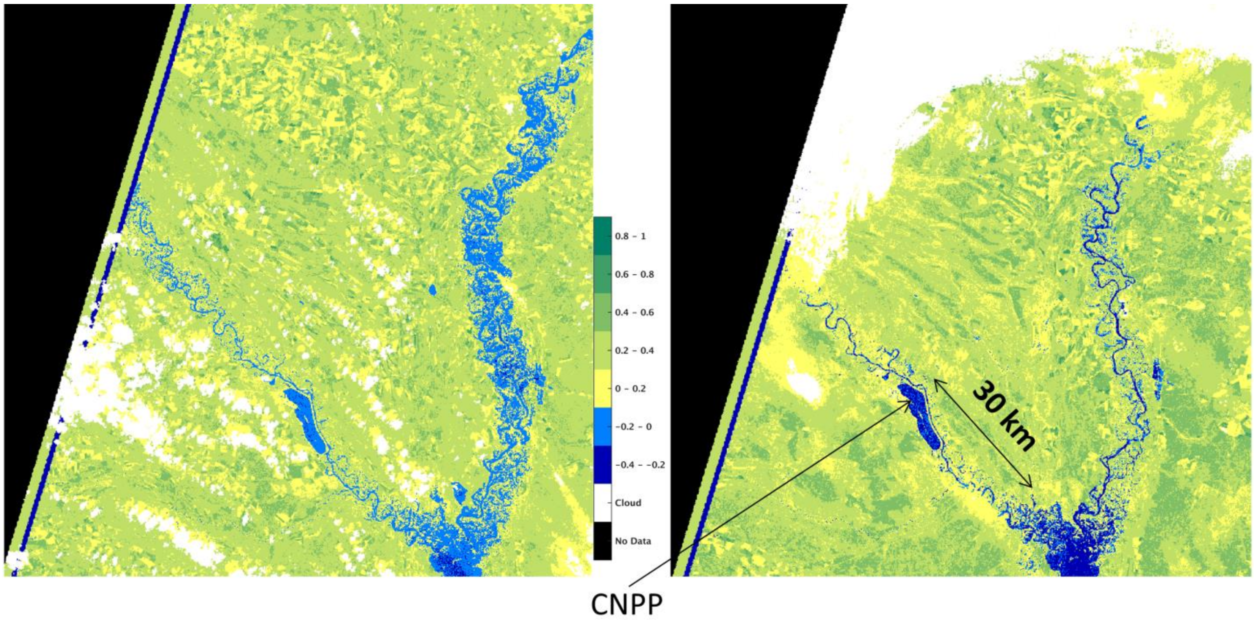

Figure S1. The Normalized Difference Vegetation Index (NDVI) maps for Chernobyl region, Ukraine and area nearby computed from LandSat 5 Thematic Mapper (TM, Bands 3 and 4) (a) before (left panel) and (b) after (right panel) CNPP accident on April 22, 1986 and April 1, 1990, respectively. The NDVI maps were created with MATLAB (version R2014b) https://www.mathworks.com/products/new_products/latest_features.html. For the color code, zero means no vegetation and close-to-one means high density of green leaves.

Reference:

1. NDVI (2000). (http://earthobservatory.nasa.gov/Features/MeasuringVegetation/measuring_vegetation_2.php)
