# Supplementary material for: External Cesium-137 doses to humans from soil influenced by the Fukushima and Chernobyl nuclear power plants accidents: a comparative study
Source: Sci Rep. 2020 May 13;10:7902. doi: 10.1038/s41598-020-64812-9 (PMC7220933; doi:10.1038/s41598-020-64812-9)
Supplement: Supplementary file 2 — Supplementary Information2. [file 41598_2020_64812_MOESM2_ESM.pdf]

Supplementary Material S2

for

External Cesium-137 doses to humans from soil influenced by the Fukushima and Chernobyl nuclear power plants accidents: a comparative study

Ka-Ming Wai[1,2,*], Dragana Krstic[3], Dragoslav Nikezic[3], Tang-Huang Lin[4], Peter K.N. Yu[5,*]

[1]Department of Civil and Environmental Engineering, College of Engineering, Shantou University, Shantou, China

[2]Intelligent Manufacturing Key Laboratory of Ministry of Education, Shantou University, Shantou, China

[3]Faculty of Science, University of Kragujevac, R. Domanovica 12, Kragujevac 34000, Serbia

[4]Center for Space and Remote Sensing Research, National Central University, Taiwan

[5]Department of Physics, City University of Hong Kong, Hong Kong SAR, China

* Corresponding Authors

E-mail: jmwei@stu.edu.cn (Ka-Ming Wai)

E-mail: peter.yu@cityu.edu.hk (Peter K.N. Yu)

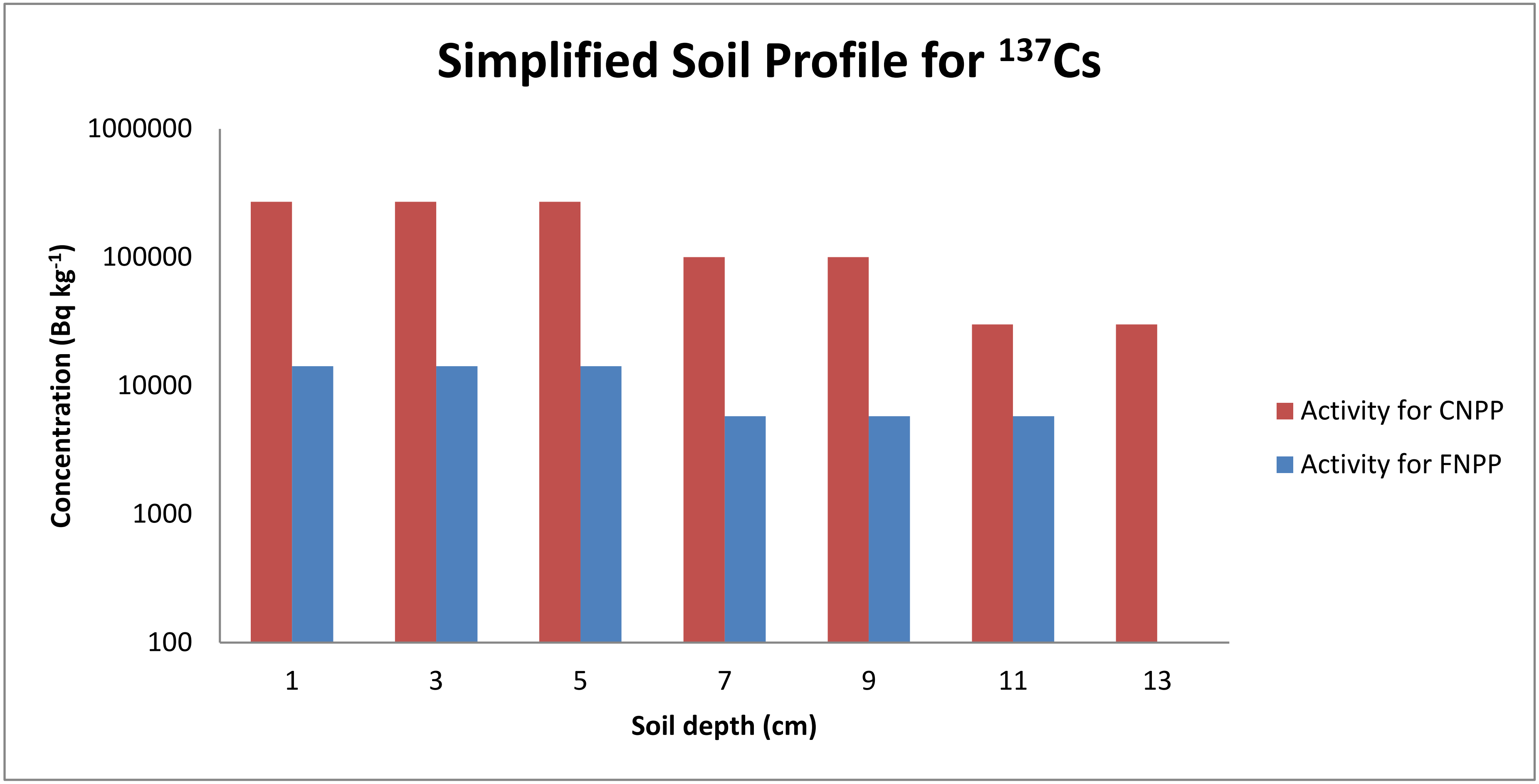
Simplified Soil Profile for 137Cs
1000000
100000
10000
1000
100
Concentration (Bq kg-1)
1
3
5
7
9
11
13
Soil depth (cm)
Activity for CNPP
Activity for FNPP
