# Supplementary material for: External Cesium-137 doses to humans from soil influenced by the Fukushima and Chernobyl nuclear power plants accidents: a comparative study
Source: Sci Rep. 2020 May 13;10:7902. doi: 10.1038/s41598-020-64812-9 (PMC7220933; doi:10.1038/s41598-020-64812-9)
Supplement: Supplementary file 3 — Supplementary Information3. [file 41598_2020_64812_MOESM3_ESM.pdf]

Supplementary Material S3

for

External Cesium-137 doses to humans from soil influenced by the Fukushima and Chernobyl nuclear power plants accidents: a comparative study

Ka-Ming Wai[1,2,*], Dragana Krstic[3], Dragoslav Nikezic[3], Tang-Huang Lin[4], Peter K.N. Yu[5,*]

[1]Department of Civil and Environmental Engineering, College of Engineering, Shantou University, Shantou, China

[2]Intelligent Manufacturing Key Laboratory of Ministry of Education, Shantou University, Shantou, China

[3]Faculty of Science, University of Kragujevac, R. Domanovica 12, Kragujevac 34000, Serbia

[4]Center for Space and Remote Sensing Research, National Central University, Taiwan

[5]Department of Physics, City University of Hong Kong, Hong Kong SAR, China

* Corresponding Authors

E-mail: jmwei@stu.edu.cn (Ka-Ming Wai)

E-mail: peter.yu@cityu.edu.hk (Peter K.N. Yu)

Conversion coefficients in various organs as a function of soil depth

Soil depth (cm)	Bone surface	Bone marrow	Skin	Gonads	Breasts	Lungs	Thyroid	Liver	Bladder	Colon	Stomach	Esophagus	Remainder
1	14.4	4.13	41.05	3.22	1.75	1.47	0.5	2.56	3.51	2.61	2.13	1.06	5.67
3	11.1	2.74	17.54	2.5	1.44	1.06	0.335	1.91	2.71	1.95	1.56	0.758	3.43
5	7	1.95	12.96	1.94	1.12	0.793	0.236	1.39	2.02	1.42	1.11	0.564	2.23
7	4.66	1.42	5.95	1.44	0.9	0.593	0.17	0.965	1.46	1.02	0.812	0.362	1.52
9	3.17	1.02	3.85	1.04	0.652	0.415	0.145	0.705	1.05	0.718	0.586	0.319	1.07
11	2.18	0.735	2.75	0.74	0.508	0.296	0.109	0.5	0.785	0.53	0.436	0.226	0.742
13	1.51	0.522	1.685	0.511	0.393	0.215	0.0612	0.477	0.575	0.393	0.296	0.138	0.524
15	1.12	0.389	1.24	0.423	0.287	0.145	0.0428	0.276	0.394	0.285	0.213	0.116	0.335
17	0.784	0.284	1.0575	0.304	0.21	0.111	0.0328	0.192	0.303	0.197	0.164	0.0836	0.261
19	0.548	0.208	0.7	0.236	0.151	0.0769	0.0253	0.139	0.218	0.139	0.123	0.061	0.192
